# Influence of carboxymethyl cellulose and calcium-crosslinked CMC coating of 3D-Printed hydroxyapatite scaffolds on the early hemostatic performance and osteoblastic compatibility

**DOI:** 10.3389/fbioe.2026.1866258

**Published:** 2026-09-04

**Authors:** Attapong Deedklin, Jintamai Suwanprateeb, Araya Pisek, Chantida Pawaputanon Na Mahasarakham, Poonsak Pisek

**Affiliations:** 1 Department of Preventive Dentistry, Faculty of Dentistry, Khon Kaen University, Khon Kaen, Thailand; 2 Department of Preventive Dentistry, Faculty of Dentistry, Naresuan University, Phitsanulok, Thailand; 3 Biofunctional Materials and Devices Research Group, National Metal and Materials Technology Center (MTEC), National Science and Technology Development Agency (NSTDA), Pathum Thani, Thailand; 4 Thammasat University Center of Excellence in Computational Mechanics and Medical Engineering, Thammasat University, Pathum Thani, Thailand; 5 Department of Restorative Dentistry, Faculty of Dentistry, Khon Kaen University, Khon Kaen, Thailand; 6 Walailak University International College of Dentistry, Bangkok, Thailand

**Keywords:** 3D-printed scaffold, blood–material interactions, calcium-crosslinked carboxymethyl cellulose, hydroxyapatite scaffold, surface modification

## Abstract

**Introduction:**

Successfully managing bl eeding bone defects relies on biomaterials capable of promoting early hemostasis alongside osteogenesis. While 3D-printed hydroxyapatite (3DP-HA) scaffolds possess osteoconductivity, their clinical utility can be limited by poor blood stability and inadequate initial hemostatic properties. To address these challenges, this preliminary study investigates the surface modification of 3DP-HA scaffolds using carboxymethyl cellulose (CMC) and its calcium-crosslinked derivative (CMC–Ca).

**Materials and methods:**

Scaffolds were coated with either 1% or 2% CMC, or a CMC–Ca complex at varying formulations (1:1, 1:2, and 2:1). Total porosity and mean pore diameter were evaluated via micro-computed tomography (micro-CT). Surface morphology and elemental properties were characterized via scanning electron microscopy (SEM) and energy-dispersive X-ray spectroscopy (EDX). Early *in vitro* hemostatic tendencies were evaluated through blood absorption and whole-blood clotting index (BCI) assays. Preliminary cytocompatibility and early osteogenic response were assessed by observing cell attachment via SEM and measuring alkaline phosphatase (ALP) activity at Day 7 using a human fetal osteoblast cell line (hFOB 1.19).

**Results:**

All modified formulations maintained total porosity ranging from 54.08% to 61.85%. Blood absorption capacity was comparable among all groups without statistically significant differences. For the *in vitro* clotting assay, the 2:1 CMC–Ca formulation exhibited a lower blood clotting index value than the other scaffold groups, suggesting a favorable initial coagulation response under the tested conditions. *In vitro* biological assays provided early descriptive evidence that the coatings permitted cell attachment and spreading by Day 7. Furthermore, ALP activity remained comparable across all groups, indicating that the surface modifications did not inherently suppress early-stage osteogenic signaling at the investigated time point.

**Conclusion:**

This preliminary study demonstrates that surface-modified 3DP-HA scaffolds maintained macro-porosity while the addition of CMC and CMC–Ca coatings provided early indications of *in vitro* hemostatic potential and early osteogenic compatibility. In particular, the 2:1 CMC–Ca formulation showed a positive initial blood clotting tendency without descriptive impairment of osteoblastic function. While further comprehensive, higher-powered quantification and mechanistic assays are required, these initial results offer a potential baseline strategy for developing surface-modified bone grafts.

## 1 Introduction

The clinical management of bone defects, which can arise from trauma, tumor resection, or congenital abnormalities, presents a significant challenge in orthopedic and craniofacial surgery. Effective treatment requires not only restoration of structural integrity but also rapid hemostasis in bleeding bone defects (Avanzi et al., 2023; Chelu and Musuc, 2023; Cui et al., 2023). Hydroxyapatite (HA), a bioceramic structurally similar to the mineral phase of natural bone, has been extensively studied for its biocompatibility and its ability to support osteoconductivity for bone regeneration (De Pace et al., 2025). Furthermore, advances in 3D printing have enabled the fabrication of patient-specific scaffolds with tailored architectures designed to support the ingrowth of bone cells and blood vessels (Khan et al., 2024). Several studies have investigated 3D-printed hydroxyapatite scaffolds (3DP-HA), highlighting their potential for bone repair applications (Kim et al., 2019; Suwanprateeb et al., 2010; Taecha-apaikun et al., 2021; Turnbull et al., 2018; Zeng et al., 2018). However, despite these structural advantages, a major limitation of conventional 3DP-HA scaffolds is their minimal intrinsic hemostatic performance. Because they primarily act as passive osteoconductive frameworks, they lack active surface functionalities capable of initiating rapid coagulation at bleeding bone surfaces, which can lead to prolonged bleeding, localized hematoma formation, or sub-optimal early-stage healing outcomes (Mirshafiei et al., 2024).

To address these limitations, surface modification offers a viable strategy to introduce functional properties without altering the interconnected porous architecture of the underlying scaffolds (Bukatuka et al., 2025; Tang et al., 2024). Numerous studies have reported that modifying HA with polymers such as chitosan, polycaprolactone, collagen, and cellulose can improve the architecture, mechanical properties, and biocompatibility of the resulting composites (Kane et al., 2015; Nikpour et al., 2012; Azevedo et al., 2003; Daugela et al., 2018; Liu et al., 2022). Surface coating and functionalization strategies have likewise been applied to HA-containing and 3D-printed scaffolds to tailor their surface morphology and biological response without compromising the underlying porous architecture (Chi et al., 2022; Mohammed et al., 2023; Ignjatović et al., 2016). Similarly, in the development of dedicated hemostatic biomaterials, researchers have frequently utilized materials like chitosan, kaolin, and collagen sponges to enhance clotting performance (Chen et al., 2020a; Hu et al., 2018; Tamer et al., 2021; He et al., 2021).

Among these modifiers, carboxymethyl cellulose (CMC) is widely used for HA alteration (Wongsawichai et al., 2019; Aminatun et al., 2020; Karatas et al., 2024). As a water-soluble, hydrophilic cellulose derivative, CMC has gained significant attention in biomaterial applications due to its high fluid absorbency and capacity for hydrogel formation (Kim et al., 2018; Liu et al., 2018; Nan et al., 2019; Rahman et al., 2021; Wang et al., 2020; Yao et al., 2024). When exposed to blood, CMC facilitates rapid fluid uptake, theoretically concentrating cellular elements and clotting factors locally—a mechanism commonly utilized in established carbohydrate-based hemostatic agents. However, native CMC dissolves rapidly in aqueous environments, presenting a challenge for maintaining structural stability and coating integrity under physiological flow conditions (Wang et al., 2020). To control this dissolution rate and optimize matrix stability, ionic crosslinking of the carboxymethyl groups using divalent calcium ions represents a practical modification strategy. Beyond its structural role in regulating hydrogel swelling, calcium serves as a critical cofactor (Factor IV) in multiple stages of the blood coagulation cascade, potentially accelerating thrombin generation and early clot stabilization (Yao et al., 2024; Zhao et al., 2024; Arumughan et al., 2022).

Consequently, combining CMC and its calcium-crosslinked form (CMC–Ca) with a 3DP-HA core scaffold presents a compelling, dual-functional rationale. The underlying HA framework provides the macro-porous architecture and chemical cues required for long-term osteoblastic activity, while the temporary CMC-based surface coating is designed to target the acute phase of bone-defect management by modulating early fluid absorption and initial blood clotting tendencies.

This combination was selected to address a gap that existing materials only partially cover. Established carbohydrate-based hemostats, such as oxidized cellulose and chitosan, are typically deployed as soft dressings or powders for compressible soft-tissue bleeding rather than as adherent coatings on a rigid, porous ceramic intended to remain within a bone defect (Kim et al., 2018; Yao et al., 2024; Zhao et al., 2024; Arumughan et al., 2022; Basagaoglu Demirekin et al., 2015; Kanjanamekanant et al., 2025; Fan et al., 2016). This study sought to combine two functions, rapid fluid uptake and local calcium availability as a coagulation cofactor, at the interface where early hemostasis occurs, while the 3DP-HA core retains its osteoconductive architecture. To our knowledge, calcium-crosslinked CMC has not previously been systematically examined as a tunable, hemostatically oriented coating on 3DP-HA scaffolds, in which CMC concentration and the CMC-to-calcium ratio are varied to modulate early blood absorption, whole-blood clotting profiles, and the early osteogenic response.

Therefore, this preliminary study aimed to investigate the effect of CMC and calcium-crosslinked CMC (CMC–Ca) coatings on *in vitro* hemostatic behavior and early osteoblastic response of 3DP-HA scaffolds. Scaffolds coated with varying CMC concentrations and CMC–Ca ratios were systematically characterized and evaluated for porosity, blood absorption capacity, whole-blood coagulation tendencies, descriptive cell attachment, and early alkaline phosphatase (ALP) activity. We hypothesized that the application of specific CMC–Ca coating formulations could improve initial blood clotting tendencies *in vitro* without compromising the early cytocompatibility and osteoblastic signaling of the underlying HA scaffold.

## 2 Materials and methods

### 2.1 Three-dimensional printed hydroxyapatite (3DP-HA) Scaffold fabrication

The 3DP-HA scaffolds were fabricated via powder-based binder jetting at the National Metal and Materials Technology Center (MTEC, Thailand), adapting previously reported methods (Turnbull et al., 2018). Using a ProJet 160 3D printer (3D Systems, United States), a water-based binder (VisiJet PXL Clear) was selectively printed onto calcium sulfate hemihydrate powder (VisiJet PXL Core) layer-by-layer to construct disc-shaped scaffolds with 8.0 mm diameter and 2.0 mm height from a CAD model.

After printing, the samples remained in the build chamber for approximately 2 h, followed by a 24-h drying period at room temperature. Compressed air was then used to clear away any loose powder. To convert the calcium sulfate precursors into hydroxyapatite, the scaffolds were incubated in a 1 M disodium hydrogen phosphate (Na_2_HPO_4_) solution (Sigma-Aldrich, United States) at 100 °C for 24 h. Finally, the converted scaffolds were thoroughly washed with deionized water and dried in an oven at 60 °C.

### 2.2 Selection of coating parameters

#### 2.2.1 CMC concentration selection

Preliminary screening demonstrated that CMC solutions below 1% (w/v) provided insufficient surface coverage on the scaffolds. Conversely, concentrations exceeding 2% (w/v) resulted in a highly viscous solution that completely occluded the internal macropores of the scaffolds, preventing uniform coating infiltration. This behavior aligns with findings by Sayed et al. (Sayed et al., 2018), who reported that the elevated viscosity of a 2.0% (w/v) CMC solution significantly hinders its diffusion throughout hydroxyapatite (HA) scaffolds. Therefore, CMC concentrations of 1% and 2% (w/v) were selected as the optimal window to ensure both adequate surface coating and the preservation of internal macroporosity.

#### 2.2.2 Calcium-crosslinked CMC ratio selection

To systematically investigate how varying the availability of crosslinking calcium ions (Ca^2+^) relative to the hydrophilic polymer matrix influences early coagulation kinetics and fluid absorption, specific calcium-to-CMC volumetric crosslinking ratios were evaluated. This range was further informed by Nan et al. (Nan et al., 2019), who demonstrated that the gel content of CMC hydrogels increases rapidly at calcium concentrations below 2%, but gradually decreases above this threshold due to saturation effects or structural transitions.

Consequently, the integration of these specific CMC concentrations (1% and 2% w/v) and crosslinking ratios (1:1, 1:2, 2:1) provides a robust framework to evaluate the interplay between coating morphology, mechanical stability, and biological performance.

### 2.3 Preparation of 3DP-HA scaffolds coated with carboxymethyl cellulose (CMC) and calcium-crosslinked carboxymethyl cellulose (CMC-Ca)

This study investigated six distinct scaffold groups: uncoated, 1% CMC, 2% CMC, 1:1 CMC-Ca, 1:2 CMC-Ca, and 2:1 CMC-Ca.

To prepare the CMC-coated scaffolds, 1% and 2% (w/v) CMC solutions were prepared by dissolving carboxymethyl cellulose powder (molecular weight 6,400, viscosity 2,200 cP, pharmaceutical grade, China) in deionized water under continuous stirring. The 3DP-HA scaffolds were immersed in the respective CMC solutions for 24 h, after that they were removed and air-dried for an additional 24 h.

For the calcium-crosslinked CMC (CMC-Ca) groups, a 1% (w/v) calcium chloride solution was mixed with either 1% or 2% (w/v) CMC solutions to produce the 1:1 and 2:1 CMC-Ca formulations, respectively. For the 1:2 CMC-Ca group, a 1% (w/v) CMC solution was combined with a 2% (w/v) calcium chloride solution. The 3DP-HA scaffolds were immersed in the corresponding coating solutions for 24 h and subsequently air-dried for a further 24 h. Uncoated scaffolds were prepared without any surface coating.

For all quantitative assessments, three independently prepared scaffold specimens per group (n = 3/group) were tested for each condition, including uncoated, 1% CMC, 2% CMC, 1:1 CMC-Ca, 1:2 CMC-Ca, and 2:1 CMC-Ca.

### 2.4 Quantitative evaluation of coating mass (weight gain percentage)

To determine the quantity of CMC and CMC–Ca matrices deposited onto the 3DP-HA scaffolds, a gravimetric analys is was performed. Prior to the surface modification process, all 3DP-HA scaffolds were dried in a vacuum oven at 60 °C until a constant mass was achieved. The initial dry weight of each individual scaffold was recorded (W_i_)​.

After coating and drying to constant mass as described above, each scaffold was reweighed (W_d_). The percentage weight gain was calculated as:

$$\text{Weight gain}\left(\%\right)=\frac{W_{d}-W_{i}}{W_{i}}x100$$

where W_i_ is the initial weight and W_d_ is the dry weight after coating.

### 2.5 Surface morphology and elemental analysis

The surface morphology and elemental composition of the 3DP-HA scaffolds from all experimental groups were evaluated to assess the coating characteristics. Prior to analysis, the samples were dried overnight at room temperature and sputter-coated with gold. Morphological imaging was performed using scanning electron microscopy (SEM; Hitachi S-3800 N, Japan) at an accelerating voltage of 5 keV. Subsequently, elemental composition was analyzed on the same instrument utilizing energy-dispersive X-ray spectroscopy (EDX) at an accelerating voltage of 20 keV. The relative atomic percentages of calcium (Ca), phosphorus (P), and sodium (Na) were quantified, from which the calcium-to-phosphorus (Ca/P) atomic ratios were derived.

### 2.6 Micro-CT porosity and structural analysis

The porosity of the scaffolds was evaluated using a high-energy micro-computed tomography (micro-CT) system (SkyScan 1,273; Bruker, Belgium). Scans were conducted over approximately 60 min utilizing a 3,072 × 1944 pixel detector, a 360° rotation, and a step size of 0.18°. The operating parameters were set to 100 kV and 80 μA, with an isotropic voxel size of 6.0 µm and a combined 1.0 mm aluminum/0.038 mm copper filter. Following acquisition, raw projection data were reconstructed using NRecon software (v1.7.0.4; Bruker). For quantitative analysis, a central volume of interest (VOI) spanning the entire height of the scaffold was defined in CTAn software (v1.24.0.0; Bruker). Greyscale images were binarised using a fixed global threshold applied identically across all samples, followed by despeckling to remove isolated voxels. In addition to total porosity and mean pore diameter, the segmented data were used to derive the open and closed porosity fractions. As the analysis was performed on a slice-resolved (2D) basis, these parameters were additionally evaluated as a function of position along the scaffold depth to assess spatial homogeneity. Data are reported as the global VOI value and, where indicated, as the mean ± SD across analysed slices.

### 2.7 Human whole blood acquisition

Human whole blood was collected from a defined cohort of healthy volunteers (n = 3; male; age range, 18–35 years) at the Khon Kaen University Blood Bank. All participants provided informed consent. Blood was drawn into tubes containing 3.8% sodium citrate as an anticoagulant. To account for inter-donor variability, samples were explicitly not pooled; rather, they were maintained and analyzed separately to accurately monitor individual baseline coagulation trends. Additionally, the use of human whole blood in this study was approved by the Khon Kaen University Ethics Committee on Human Research (Approval No. HE661594).

### 2.8 Blood absorption capacity

The maximum blood absorption capacity was assessed by immersing samples from each group into 500 μL of human whole blood. Each sample was first weighed to establish its initial weight (W_0_​). After immersion, the samples were removed and weighed again (W_1_) at 30, 60, and 180 s to measure the amount of blood absorbed. The blood absorption capacity was then calculated using the following equation:

$$\text{Blood absorption capacity}\left(\%\right)=\frac{W_{1}}{W_{0}}x100$$

where W_0_ is the initial weight and W_1_ is the weight after blood absorption.

### 2.9 Whole-blood clotting *in vitro*

The blood coagulation behavior of the scaffolds was evaluated using a Blood Clotting Index (BCI) assay. First, the scaffolds were placed at the bottom of the test tubes. Subsequently, a mixture containing 200 µL of anticoagulated human whole blood and 200 µL of 0.2 mol L^-1^ CaCl_2_ was swiftly added to each tube. To validate the assay, A positive control group, consisting of 200 µL of human whole blood with 200 µL of 0.2 mol L^-1^ CaCl_2_, and a negative control group, containing only 400 µL of anticoagulated blood, were prepared simultaneously. All test tubes were then incubated at 37 °C with shaking.

At specified time intervals (30 s, 60 s, and 180 s), 10.0 mL of deionized water was slowly added to separate the uncoagulated erythrocytes. The resulting supernatant was collected, and its absorbance was measured at 540 nm using a UV-Vis spectrophotometer (Varioskan LUX, Thermo Fisher Scientific Oy Ratastie 2, Finland). Finally, the BCI was calculated according to the following equation to determine the hemostatic efficacy of each scaffold:

$$\text{Blood clotting index}\left(\%\right)=\frac{A_{s}}{A_{0}}x100$$

where A_s_ is the absorbance of the supernatant of the sample group and A_0_ is the absorbance of the supernatant of the negative control group.

### 2.10 Cell culture

The human fetal osteoblast cell line hFOB 1.19 (ATCC Cat# CRL-11372, RRID:CVCL_3708; American Type Culture Collection) was used. Cells were cultured in a 1:1 mixture of Ham’s F-12 and Dulbecco’s Modified Eagle’s Medium, supplemented with 2.5 mM L-glutamine (without phenol red), 10% fetal bovine serum, and 0.3% gentamicin (G418). The cells were incubated at 37 °C in a humidified atmosphere containing 5% CO_2_. Upon reaching approximately 80% confluence, the cells were rinsed with phosphate-buffered saline (PBS), detached using 0.2% trypsin–EDTA, and subsequently seeded onto the scaffolds for evaluation of cell attachment and alkaline phosphatase activity.

### 2.11 Cell attachment on scaffolds

To evaluate cell attachment on the scaffolds, all scaffolds were sterilized with UV light for 30 min. The scaffolds were then pre-soaked in a cell culture medium for 30 min to facilitate full absorption, and any excess medium was removed. Human fetal osteoblast cells (hFOB1.19) were seeded onto the scaffolds at a density of 50,000 cells per well. The samples were divided into eight groups: 1CMC scaffolds, 2CMC scaffolds, 1:1 CMC-Ca scaffolds, 1:2 CMC-Ca scaffolds, 2:1 CMC-Ca scaffolds, uncoated scaffolds, a positive control group, which is cells cultured in DMEM/Ham’s F12 (1:1) without scaffolds, and a negative control group, which is cells cultured with 10% dimethyl sulfoxide (DMSO).

After incubation for 7 days, the cell-scaffold constructs from each group were transferred to a new 24-well plate. The scaffolds were rinsed with phosphate-buffered saline (PBS) and then fixed for 1 hour at room temperature using a 4% glutaraldehyde solution. After fixation, the glutaraldehyde was removed, and the samples were dried at room temperature for 24 h. The dried samples were then sputter-coated with gold and examined using a scanning electron microscope (Hitachi S-3000 N, Tokyo, Japan) with an accelerating voltage of 5 keV.

### 2.12 Alkaline phosphatase activity

To evaluate alkaline phosphatase (ALP) activity, all scaffolds were sterilized with UV light for 30 min, pre-soaked in cell culture medium for 30 min to facilitate full absorption, and cleared of excess medium. Human fetal osteoblast cells (hFOB 1.19) were then seeded onto the scaffolds at a density of 50,000 cells per well.

After 7 days of incubation, the cell culture medium was collected from each group to evaluate secreted ALP activity. An alkaline phosphatase activity estimation kit (EZAssay CCK035, HiMedia, India) was used to measure ALP levels in the collected medium. 20 μL of the medium from each sample was mixed with the prepared buffer and substrate according to the manufacturer’s instructions. The change in absorbance at 405 nm, which is directly proportional to ALP activity, was recorded after 30 min using a microplate reader.

To control for serum-derived protein interference from the 10% FBS in the growth medium, unseeded medium incubated under identical conditions was used as a blank baseline for both the ALP activity assay and the subsequent Bradford protein assay. This background absorbance was systematically subtracted from all experimental sample values to ensure that the quantified protein and enzyme activities originated solely from cellular secretion. Finally, the corrected ALP activity was normalized to the corrected total protein content.

### 2.13 Statistical analysis

Data are reported using the mean and standard deviation. All quantitative assays were performed with three independent scaffold specimens per group. Statistical analyses were conducted using SPSS (IBM Corp., Armonk, NY, United States). The Shapiro-Wilk test was used to assess the normality of the data distribution.

Differences in all measurements among groups were evaluated using a one-way analysis of variance (ANOVA), followed by Tukey’s *post hoc* test for pairwise comparisons. A p-value <0.05 was considered statistically significant.

## 3 Results

### 3.1 Weight gain percentage

To quantitatively evaluate the deposition of the polymeric matrix onto the 3DP-HA scaffolds, the percentage weight gain after the coating process was calculated (Table 1).

**TABLE 1:** Percentage weight gain of 3DP-HA scaffolds following surface modification with CMC and CMC–Ca coating formulations. Data are presented as Mean ± SD (n = 3).

Samples	Weight gain (%)
1 CMC	2.13 ± 1.46
2 CMC	9.07 ± 4.76
1:1 CMC-Ca	6.59 ± 1.63
1:2 CMC-Ca	4.72 ± 1.92
2:1 CMC-Ca	9.74 ± 2.49
Uncoated	0.00 ± 0.00

Among the CMC formulations, the 1CMC exhibited a minor weight gain of 2.13% ± 1.46%, while 2CMC resulted in a higher descriptive mass deposition of 9.07% ± 4.76%. For the calcium-crosslinked formulations, the coating mass appeared to be influenced by the specific polymer-to-crosslinker ratios. The 1:2 CMC–Ca and 1:1 CMC–Ca groups yielded weight gain values of 4.72% ± 1.92% and 6.59% ± 1.63%, respectively. Notably, the 2:1 CMC–Ca formulation displayed the highest descriptive mass accumulation among the crosslinked groups, reaching 9.74% ± 2.49%.

### 3.2 Surface morphology

**FIGURE 1:**
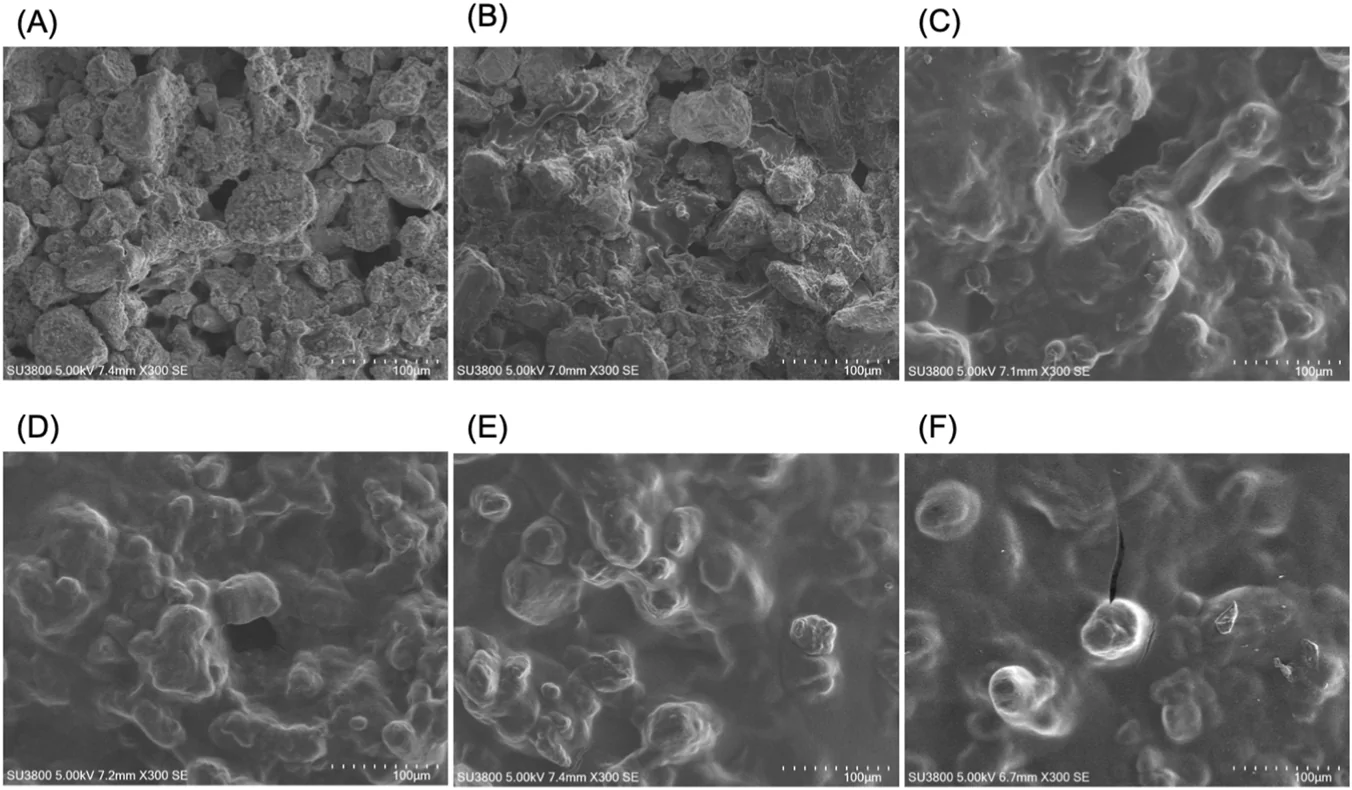
Scanning electron microscopy (SEM) showing the surface morphology of the scaffolds. Images were acquired on a Hitachi S-3800 N scanning electron microscope at an accelerating voltage of 5 keV and a magnification of ×300: **(A)** Uncoated, **(B)** 1 CMC, **(C)** 2 CMC, **(D)** 1:1 CMC-Ca, **(E)** 1:2 CMC-Ca, and **(F)** 2:1 CMC-Ca.

Scanning electron microscopy revealed clear differences in surface morphology among the scaffold groups (Figure 1). The uncoated scaffold (Figure 1A) exhibited a highly irregular and porous surface composed of loosely packed, angular hydroxyapatite particles. In contrast, all coated scaffolds displayed noticeably smoother surfaces. The 1% CMC–coated scaffold (Figure 1B) showed a rough and granular morphology with a thin, discontinuous coating, leaving individual HA particles clearly visible. Increasing the CMC concentration to 2% (Figure 1C) resulted in a smoother and less granular surface with a more continuous coating layer. Scaffolds coated with CMC-Ca exhibited a smooth and continuous gel-like surface. The 1:1 (Figure 1D) and 1:2 CMC-Ca–coated scaffolds (Figure 1E) showed uniform coatings, although the 1:2 formulation contained more prominent embedded particles. The 2:1 CMC-Ca–coated scaffold (Figure 1F) exhibited the most homogeneous and smoothest surface, with fewer and smaller embedded particles. Overall, these observations indicate that both CMC and CMC-Ca coatings effectively modify scaffold surface morphology, with increasing calcium-to-CMC ratio producing a more uniform coating.

### 3.3 Elemental analysis (EDX)

Surface elemental analysis via EDX (Table 2) revealed that the uncoated 3DP-HA scaffolds possessed a baseline Ca/P atomic ratio of 1.83, with surface concentrations of Calcium (Ca) and Phosphorus (P) at 0.76% ± 0.09% and 0.42% ± 0.01%, respectively. Upon application of the carboxymethyl cellulose (CMC) coating, the absolute atomic percentages of Ca, P, and Sodium (Na) dropped below 1.0% across all experimental groups, as the total elemental profile became dominated by the Carbon and Oxygen matrices of the polymer. In the uncrosslinked 2 CMC group, the P signal decreased to a near-detection limit of 0.01% ± 0.01% while Na peaked at 0.69% ± 0.10%, driving the calculated surface Ca/P ratio to an anomalous 5.92. Following ionic crosslinking (CMC-Ca groups), successful ion exchange was confirmed by a sharp drop in Na concentration to approximately 0.06% ± 0.01% in the 1:1 and 1:2 formulations, which corresponded to modulated Ca/P surface atomic ratios ranging from 1.51 to 2.33.

**TABLE 2:** The percentage of elemental composition of the scaffolds from EDX (n = 3). Data are presented as the Mean ± SD and were calculated using atomic percentages (at. %).

Samples	Atomic percentage (at%)			Ca/P
	Ca	P	Na	
1 CMC	0.68 ± 0.08	0.41 ± 0.03	0.08 ± 0.01	1.66
2 CMC	0.07 ± 0.01	0.01 ± 0.01	0.69 ± 0.10	5.92
1:1 CMC-Ca	0.76 ± 0.26	0.36 ± 0.11	0.06 ± 0.01	2.11
1:2 CMC-Ca	0.36 ± 0.09	0.24 ± 0.05	0.06 ± 0.01	1.51
2:1 CMC-Ca	0.55 ± 0.12	0.23 ± 0.02	0.20 ± 0.06	2.33
Uncoated	0.76 ± 0.09	0.42 ± 0.01	0.00 ± 0.00	1.83

Analyzed elements include Ca (calcium), P (phosphorus), and Na (sodium). Ca/P represents the calcium-to-phosphorus atomic ratio.

### 3.4 Micro-CT porosity and structural analysis

**FIGURE 2:**
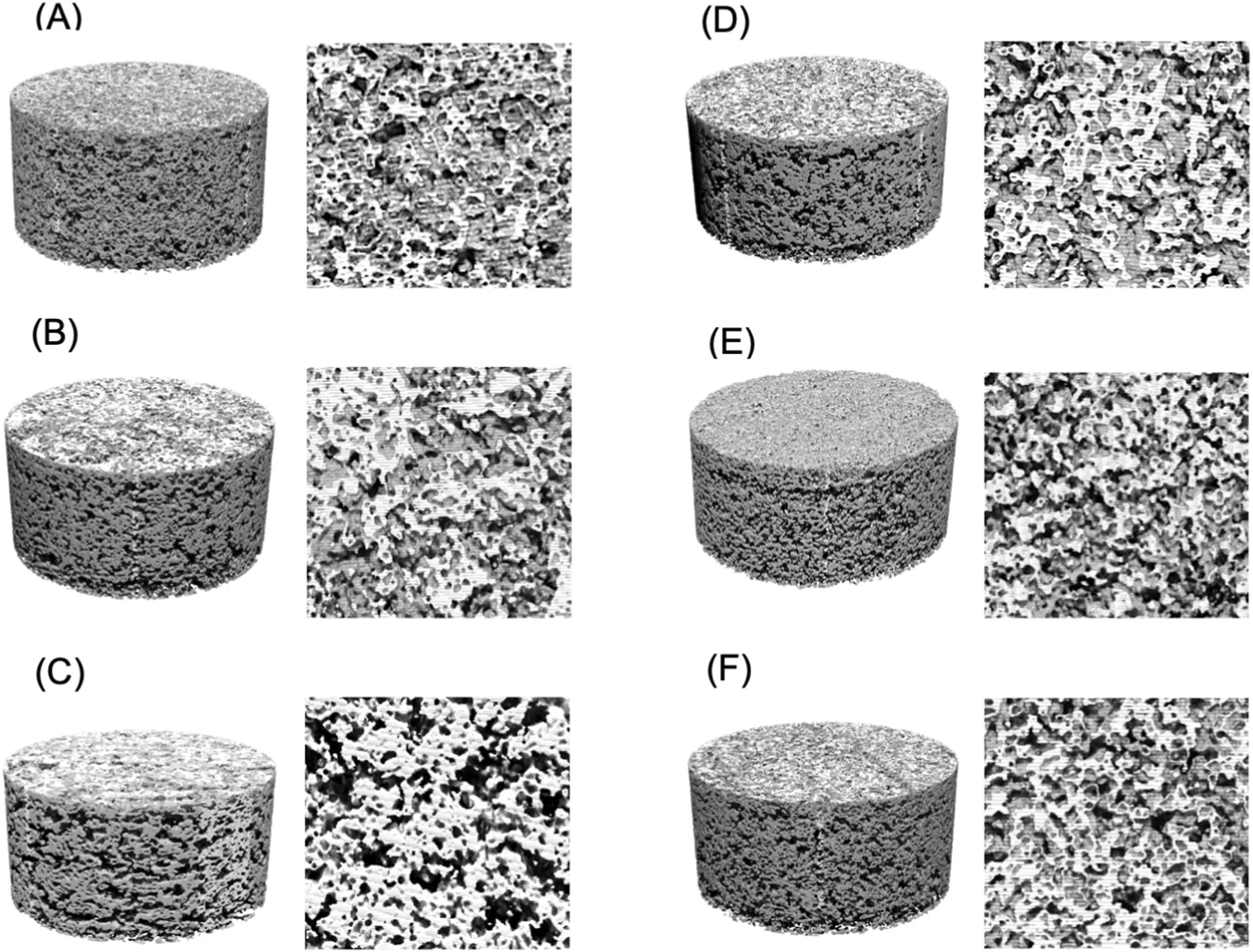
The 3D reconstructed images and cross-sections display the structural variations across the experimental groups, acquired by micro-computed tomography (SkyScan 1273, Bruker; 100 kV and 80 μA; isotropic voxel size, 6.0 µm; 1.0 mm Al/0.038 mm Cu filter; reconstructed with NRecon). Representative images of **(A)** Uncoated, **(B)** 1 CMC, **(C)** 2 CMC, **(D)** 1:1 CMC-Ca, **(E)** 1:2 CMC-Ca, and **(F)** 2:1 CMC-Ca groups.

3D reconstructions show that all scaffold formulations exhibit porous structures, though surface morphology varies across the different compositions (Figure 2). The uncoated and 1 CMC groups (Figures 2A,B) present a uniform, open porous network. Conversely, a denser surface appearance with thicker strut features and partial pore bridging is observed in the 2 CMC, 1:1 CMC-Ca, and 2:1 CMC-Ca groups (Figures 2C,D,F). Notably, the 1:2 CMC-Ca scaffold (Figure 2E) exhibits a dense polymeric layer covering its top surface.

Quantitative analysis (Table 3) confirmed significant variations in surface porosity and pore diameter across groups (p < 0.001). Compared to the uncoated control (58.18% ± 0.74%), surface porosity peaked in the 1CMC group (61.85% ± 0.64%) and dropped lowest in the 2:1 CMC-Ca group (54.08% ± 0.24%). Furthermore, thicker or crosslinked coatings reduced surface pore diameters from 26.25 ± 0.73 µm (uncoated) to 23.46 ± 0.80 µm (2CMC) and 23.91 ± 0.25 µm (2:1 CMC-Ca).

**TABLE 3:** Micro-CT analysis of scaffold porosity and pore diameter. Data are presented as Mean ± SD (n = 3).

Samples	Total porosity (%)	Open porosity (%)	Closed porosity (%)	Pore Diameter (µm)
1 CMC	61.85 ± 0.64^a^	53.29 ± 0.83^a^	8.56 ± 0.40^a^	28.01 ± 0.39^a^
2 CMC	55.91 ± 0.73^bc^	41.99 ± 1.33^b^	13.92 ± 0.61^b^	23.46 ± 0.80^d^
1:1 CMC-Ca	56.08 ± 1.43^bc^	52.16 ± 1.96^a^	3.92 ± 0.54^c^	25.56 ± 0.61^bc^
1:2 CMC-Ca	57.42 ± 1.54^b^	53.35 ± 0.19^a^	4.06 ± 1.65^c^	25.10 ± 1.04^bcd^
2:1 CMC-Ca	54.08 ± 0.24^c^	43.23 ± 1.34^b^	10.85 ± 1.11^ab^	23.91 ± 0.25^cd^
Uncoated	58.18 ± 0.74^b^	53.45 ± 1.07^a^	4.72 ± 1.80^c^	26.25 ± 0.73^ab^
p-value*	<0.001	<0.001	<0.001	<0.001
F-value	21.24	57.32	38.30	17.29
η^2^ (eta-squared)	0.90	0.96	0.94	0.88

Statistical significance was assessed by one-way ANOVA, and Tukey’s *post hoc* test. In any given column, means followed by different lowercase letters differ significantly (p < 0.05). The asterisk (*) identifies the p-value from the one-way ANOVA., η^2^ represents eta-squared as a measure of effect size. η^2^ values of 0.01, 0.06, and 0.14 indicate small, medium, and large effects, respectively (Richardson, 2011).

3D reconstructions confirm that all scaffolds maintain porosity, though surface morphology varies by composition (Figure 2). Uncoated and 1 CMC scaffolds present a uniform, open porous network. Conversely, the 2 CMC, 1:1 CMC-Ca, and 2:1 CMC-Ca groups exhibit surface densification characterized by thickened struts and partial pore bridging. The 1:2 CMC-Ca scaffold displays a dense polymeric layer covering the majority of its surface.

Quantitative analysis (Table 3) showed variations in surface porosity and pore diameter among the groups (p < 0.001). Compared to the uncoated control (58.18% ± 0.74%), the highest surface porosity value was recorded in the 1CMC group (61.85% ± 0.64%), while the lowest value was observed in the 2:1 CMC-Ca group (54.08% ± 0.24%). Furthermore, lower surface pore diameters were measured in groups with thicker or crosslinked coatings, with values of 23.46 ± 0.80 μm for 2CMC and 23.91 ± 0.25 μm for 2:1 CMC-Ca, compared to 26.25 ± 0.73 μm for the uncoated control.

### 3.5 Blood absorption capacity

**FIGURE 3:**
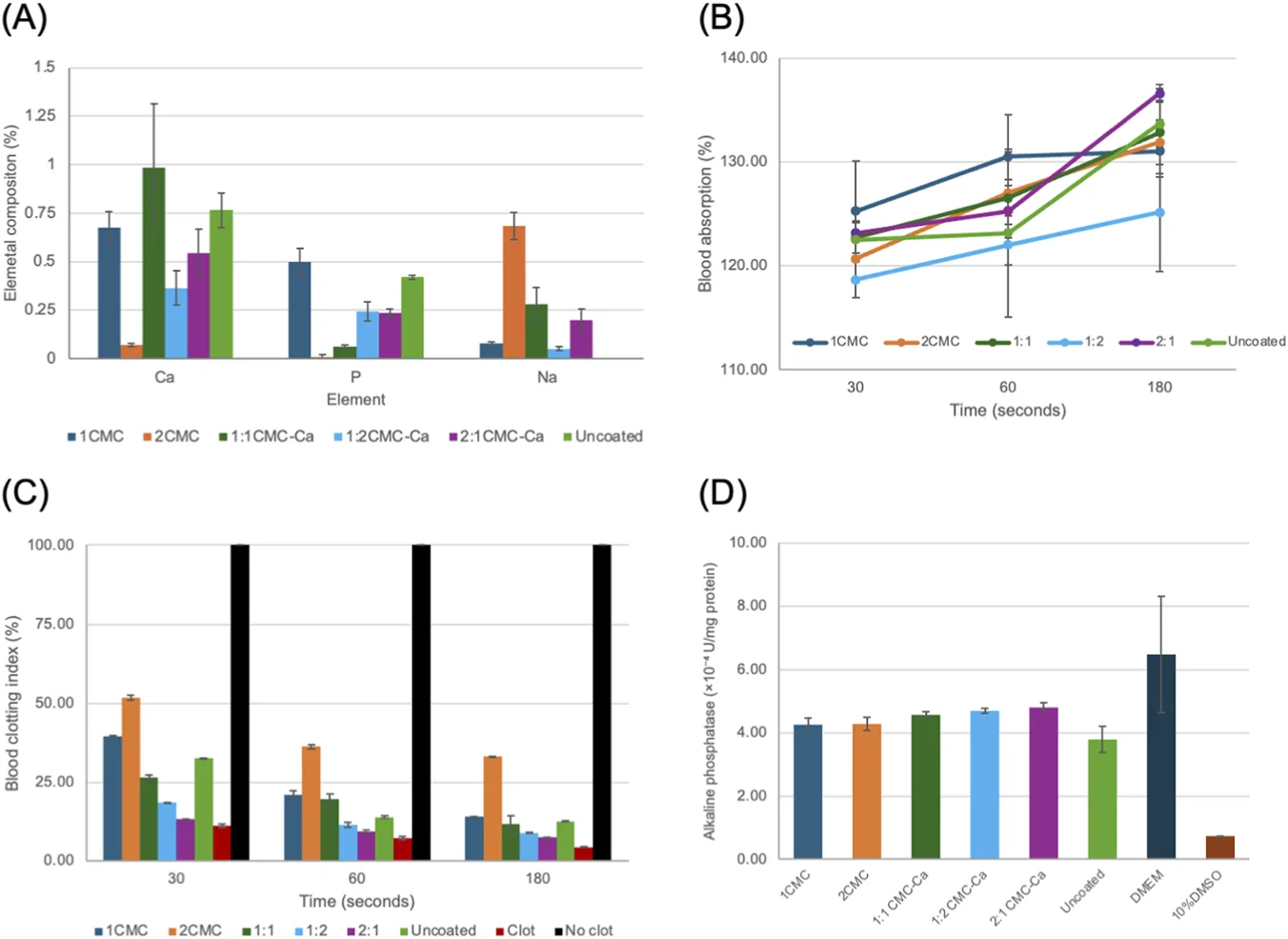
Performance analysis of 3DP-HA scaffolds coated with CMC and CMC-Ca. **(A)** Quantitative elemental analysis of the scaffold surfaces. **(B)** Blood absorption capacity monitored for 180 s. **(C)** Hemostatic potential indicated by the blood clotting index over 180 s. **(D)** Cellular osteogenic differentiation assessed via ALP activity after 7 days.

The blood absorption capacity of the scaffolds was evaluated at 30, 60, and 180 s (Table 4; Figure 3B). All scaffolds displayed blood uptake within the first 30 s, with values ranging from 118.68% ± 1.76% (1:1 CMC-Ca) to 125.42% ± 4.83% (uncoated). Blood absorption values increased slightly over time across all groups, reaching values between 125.45% ± 5.68% (1:1 CMC-Ca) and 136.57% ± 0.83% (1:2 CMC-Ca) at 180 s. However, no statistically significant differences in blood absorption capacity were observed among the formulation groups at any of the evaluated time points.

**TABLE 4:** The blood absorption percentages of each scaffold. Data are presented as Mean ± SD (n = 3).

Samples	Blood absorption capacity (%)		
	30s	60s	180s
1 CMC	120.89 ± 2.21	127.36 ± 3.92	131.86 ± 2.14
2 CMC	122.76 ± 1.45	126.59 ± 1.74	132.87 ± 4.28
1:1 CMC-Ca	118.68 ± 1.76	122.04 ± 1.94	125.45 ± 5.68
1:2 CMC-Ca	123.08 ± 2.34	125.16 ± 2.53	136.57 ± 0.83
2:1 CMC-Ca	122.50 ± 1.85	123.54 ± 8.08	133.68 ± 2.25
Uncoated	125.42 ± 4.83	130.66 ± 4.01	130.93 ± 2.18
p-value*	0.28	0.45	0.09
F-value	1.45	1.03	2.50
η^2^ (eta-squared)	0.38	0.30	0.51

Statistical significance was assessed by one-way ANOVA, and Tukey’s *post hoc* test. In any given column, means followed by different lowercase letters differ significantly (p < 0.05). The asterisk (*) identifies the p-value from the one-way ANOVA. *η* ^*2*^_*p*_ represents eta-squared as a measure of effect size. η^2^ represents eta-squared as a measure of effect size. η^2^ values of 0.01, 0.06, and 0.14 indicate small, medium, and large effects, respectively (Richardson, 2011).

### 3.6 Macroscopic assessment of whole blood coagulation

**FIGURE 4:**
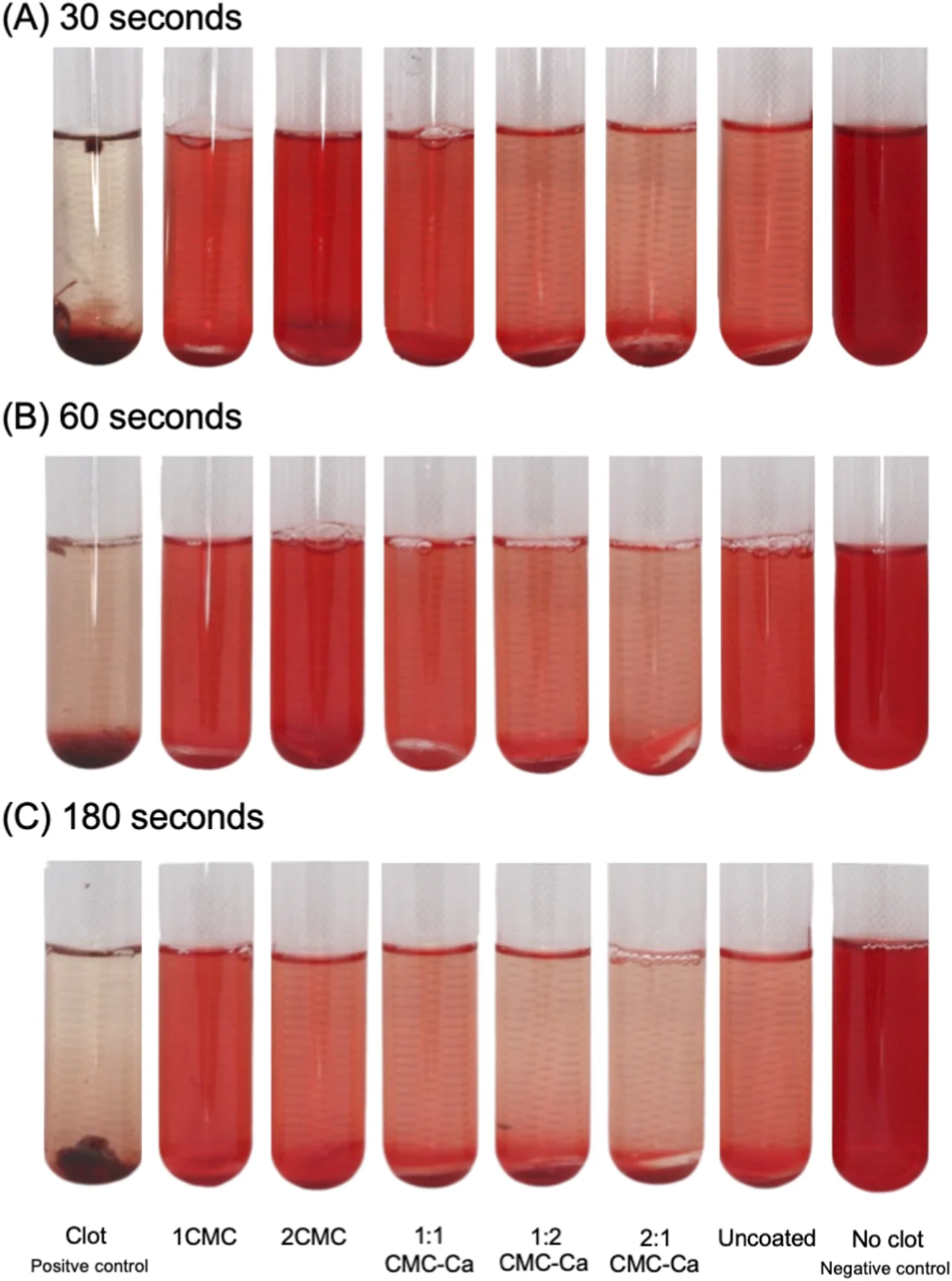
Macroscopic assessment of the whole-blood coagulation process on the different 3DP-HA scaffold groups at three time points. All tubes were imaged under uniform illumination against a neutral background at a fixed working distance with identical camera settings: **(A)** 30 s, **(B)** 60 s, and **(C)** 180 s.

Figure 4 shows representative macroscopic images of whole blood in contact with the different 3DP-HA scaffold groups at 30, 60, and 180 s, together with the positive (clot) and negative controls (no-clot). In the clot control, the supernatant cleared and a compact red pellet formed at the base of the tube from 30 s onward, consistent with coagulation and erythrocyte sedimentation. The no-clot control remained a homogeneous, intensely red suspension at all time points, with no visible clearing or pellet.

At 30 s (Figure 4A), the CMC-coated scaffolds (1CMC, 2CMC) and the uncoated scaffold retained turbid red suspensions with little visible clearing, similar to the no-clot control. The CMC-Ca-coated scaffolds, particularly the 1:2 and 2:1 formulations, showed slightly paler supernatants, suggesting modest early sedimentation relative to the other scaffold groups.

At 60 s (Figure 4B), the supernatants of the scaffold groups appeared marginally less turbid than at 30 s, consistent with gradual, time-dependent sedimentation. The 1:2 and 2:1 CMC-Ca tubes remained among the clearer scaffold groups, while the CMC-coated and uncoated groups retained more saturated red suspensions; differences among the groups were modest at the macroscopic level.

By 180 s (Figure 4C), clearing of the supernatant had progressed across all scaffold groups, indicating continued, time-dependent sedimentation. The CMC-Ca-coated scaffolds, most evidently the 1:2 and 2:1 formulations, exhibited paler supernatants than the CMC-coated and uncoated groups, whereas the latter retained more residual turbidity. Throughout, the clot and no-clot controls remained clearly distinguishable from one another and from the scaffold groups. Because the macroscopic differences among the coated and uncoated scaffolds were subtle, the quantitative blood clotting index (Table 4) was used to resolve them.

### 3.7 Blood clotting index (BCI)

The blood clotting behavior of 3DP-HA scaffolds with different CMC coatings and CMC-Ca ratios was evaluated using the blood clotting index (BCI) at 30, 60, and 180 s (Table 5; Figure 3C). Lower BCI values indicate a higher mass of formed clot under these experimental conditions.

**TABLE 5:** The percentage of the blood clotting index measured over time. Data are presented as Mean ± SD (n = 3).

Samples	Blood clotting index (%)		
	30s	60s	180s
1 CMC	39.60 ± 0.79^a^	20.86 ± 0.56^a^	14.00 ± 0.10^a^
2 CMC	51.73 ± 0.84^b^	36.23 ± 1.51^b^	33.06 ± 2.60^b^
1:1 CMC-Ca	26.50 ± 0.10^c^	19.61 ± 0.78^ac^	11.67 ± 0.10^acd^
1:2 CMC-Ca	18.46 ± 0.09^d^	11.37 ± 0.42^df^	8.76 ± 0.05^de^
2:1 CMC-Ca	13.13 ± 0.09^e^	9.35 ± 0.31^de^	7.29 ± 0.14^eg^
Uncoated	32.57 ± 0.61^f^	13.89 ± 0.56^f^	12.55 ± 0.17^acf^
Positive control	11.11 ± 0.11^g^	7.05 ± 1.27^e^	4.29 ± 0.09^g^
Negative control	100.00 ± 0.00^h^	100.00 ± 0.00^g^	100.00 ± 0.00^h^
p-value*	<0.001	<0.001	<0.001
F-value	77,737.51	2,808.84	2,379.35
η^2^ (eta-squared)	1.00	0.99	0.99

Statistical significance was assessed by one-way ANOVA, and Tukey’s *post hoc* test. In any given column, means followed by different lowercase letters differ significantly (p < 0.05). The asterisk (*) identifies the p-value from the one-way ANOVA.

The positive control group consisted of 200 µL of human whole blood with 200 µL of 0.2 mol L^-1^ CaCl_2_, while the negative control group consisted of only 400 µL of anticoagulated blood. η^2^ represents eta-squared as a measure of effect size. η^2^ values of 0.01, 0.06, and 0.14 indicate small, medium, and large effects, respectively (Richardson, 2011).

At 30 s, all scaffold groups exhibited significantly lower BCI values compared to the negative control. Mean BCI values varied among the scaffold formulations; the 2CMC group reached 51.73% ± 0.84%, whereas the 2:1 CMC-Ca group reached 13.13% ± 0.09%. The BCI values of the 2:1 CMC-Ca group were closest to those of the positive control (11.11% ± 0.11%), though this difference was statistically significant.

At 60 and 180 s, lower BCI values were recorded across all scaffold groups compared to the 30-s time point, and the numerical ranking among the formulations was similar to that observed at 30 s. At both time points, the CMC-Ca coated scaffolds exhibited lower BCI values than the CMC-coated and uncoated groups. The 2:1 CMC-Ca formulation showed the lowest mean BCI at all time points, with values of 9.35% ± 0.31% at 60 s and 7.29% ± 0.14% at 180 s. At these later time points, the BCI values for the 2:1 CMC-Ca formulation did not differ statistically from the positive control.

### 3.8 Cell attachment on scaffold

**FIGURE 5:**
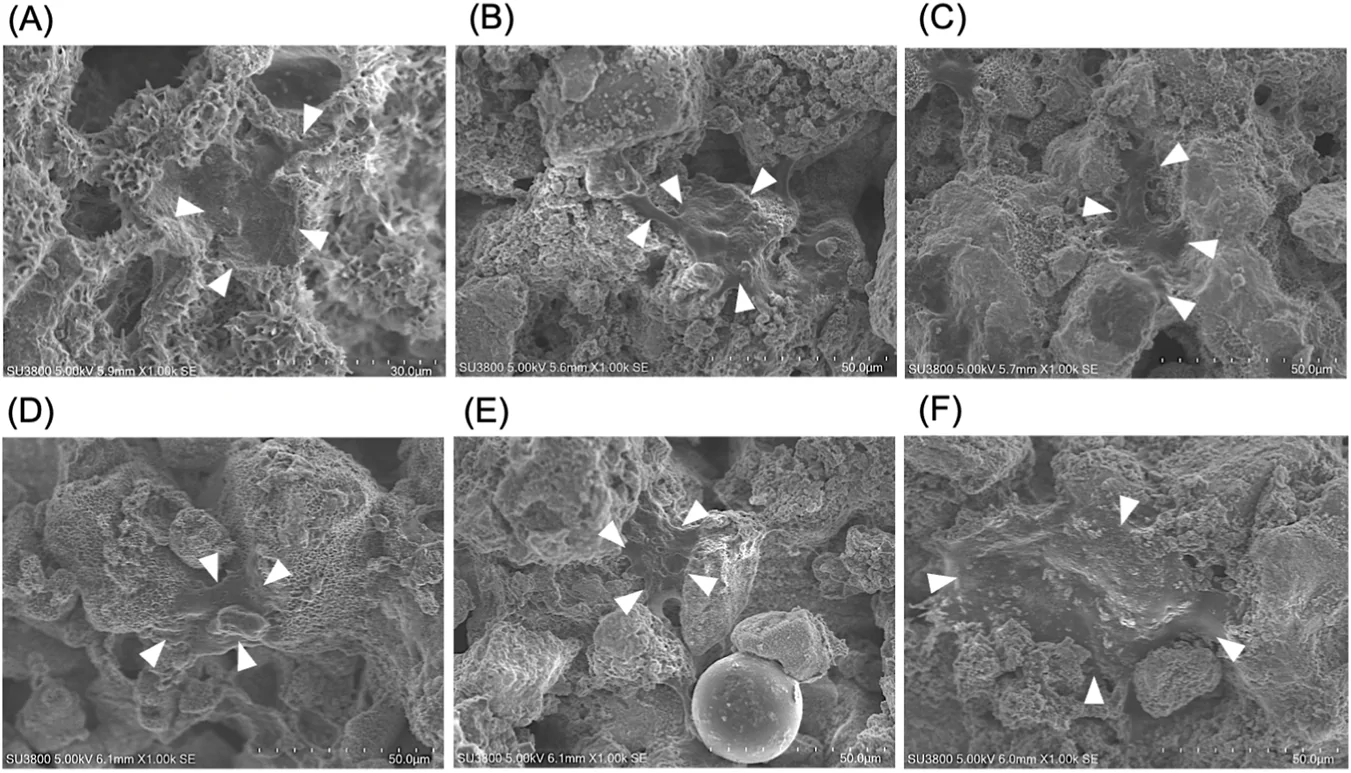
Scanning electron microscopy (SEM) micrographs (Hitachi S-3000 N; accelerating voltage, 5 keV; 1,000x) showing osteoblast morphology and attachment on 3D-printed hydroxyapatite scaffolds after 7 days of culture. Representative images of **(A)** uncoated scaffold, **(B)** 1 CMC-coated, **(C)** 2 CMC-coated, **(D)** 1:1 CMC-Ca-coated, **(E)** 1:2 CMC-Ca-coated, and **(F)** 2:1 CMC-Ca-coated. The white arrows indicate osteoblast cells attached to the scaffold surfaces.

Figure 5 presents representative SEM images of hFOB 1.19 cells cultured on coated and uncoated 3DP-HA scaffolds after 7 days. Cell attachment was observed on all scaffold surfaces, indicating that none of the coating formulations inhibited cell adhesion.

SEM micrographs showed that cells exhibited a spread, flattened morphology with multiple cytoplasmic extensions across the scaffold surfaces. No apparent qualitative differences in cell density or morphology were observed among the coated and uncoated groups.

### 3.9 Alkaline phosphatase (ALP) activity

ALP activity at day 7 was assessed as an indicator of early osteogenic differentiation (Table 6; Figure 3D). The DMEM control exhibited the highest ALP activity (6.49 ± 2.20 ×10^−4^U/mg protein), whereas the 10% DMSO negative control showed markedly reduced ALP levels (0.73 ± 0.01 ×10^−4^U/mg protein; p < 0.001).

**TABLE 6:** The Alkaline phosphatase (ALP) activity at day 7. Data are presented as Mean ± SD (n = 3).

Samples	ALP activity (×10^−4^ U/mg protein)
1 CMC	4.28 ± 0.23^a^
2 CMC	4.30 ± 0.37^a^
1:1 CMC-Ca	4.59 ± 0.12^ab^
1:2 CMC-Ca	4.70 ± 0.10^ab^
2:1 CMC-Ca	4.82 ± 0.15^ab^
Uncoated	3.81 ± 0.41^a^
Positive control	6.49 ± 2.20^b^
Negative control	0.73 ± 0.01^c^
p-value*	<0.001
F-value	16.90
η^2^ (eta-squared)	0.88

Statistical significance was assessed by one-way ANOVA, and Tukey’s *post hoc* test. In any given column, means followed by different lowercase letters differ significantly (*p* < 0.05). The asterisk (*) identifies the p-value from the one-way ANOVA.

The positive control group consists of cells cultured in DMEM/Ham’s F12 (1:1) without scaffolds, while the negative control group consists of cells cultured with 10% dimethyl sulfoxide. η^2^ represents eta-squared as a measure of effect size. η^2^ values of 0.01, 0.06, and 0.14 indicate small, medium, and large effects, respectively (Richardson, 2011).

ALP values for all scaffold groups were comparable, ranging from 3.81 ± 0.41 to 4.82 ± 0.15 ×10^−4^U/mg protein, with no statistically significant differences among the formulations (p > 0.05). Although these values were lower than those of the DMEM control, the CMC-Ca-coated groups showed no statistically significant difference from the control.

## 4 Discussion

The present study describes how the surface modification of 3DP HA scaffolds with CMC and CMC Ca coatings appears to modulate early blood material interactions as suggested by the observed blood absorption across all groups and the initial coagulation tendencies in the CMC Ca coated scaffolds while preserving early osteoblastic compatibility.

The quantitative measurement of percentage weight gain describes the deposition of CMC and CMC-Ca matrices onto the 3DP-HA scaffolds establishing an initial physical baseline prior to functional *in-vitro* assays. The observed mass variations appear to align with trends in solution viscosity where the higher weight gain of the 2% CMC formulation compared to the 1% formulation is consistent with increased polymer concentration and potential boundary layer adherence and crosslinking dynamics where the highest descriptive weight gain in the 2:1 CMC-Ca group suggests that specific polymer-to-calcium ratios permit the retention of a more substantial composite layer. Maintaining the weight gain below approximately 10% across all groups serves as a notable structural baseline that supported by micro-CT and SEM data appears to avoid excessive physical clogging of the primary interconnected pore channels thereby preserving the macro-architecture. Nevertheless certain methodological boundaries remain in this preliminary investigation as these gravimetric measurements solely reflect overall scaffold weight changes rather than localized spatial distributions. Consequently, the precise coating amount per unit surface area, absolute coating thickness, and microstructural uniformity across the complex internal architectures were not fully quantified within the scope of this study, and future comprehensive studies, including cross-sectional SEM, FTIR, XPS, rheology, swelling, and degradation analyses, are warranted to precisely delineate these parameters.

SEM analysis exhibited distinct differences in surface morphology among the scaffold groups. Both the CMC- and CMC-Ca-coated scaffolds exhibited relatively smooth and continuous surface layers. In contrast, the 1% CMC coating formed a thinner layer with surface features closely resembling those of the uncoated scaffolds. Although the CMC and CMC-Ca coatings slightly reduced overall porosity, the scaffolds retained sufficient porosity and interconnectivity for osteoblast infiltration (Cheng et al., 2014; Chen et al., 2020b; Jiao et al., 2023). This preservation suggests that the coating process was predominantly confined to the surface.

Surface elemental analysis via EDX revealed that the uncoated 3DP-HA scaffolds possessed dominant calcium (Ca) and phosphorus (P) signals characteristic of hydroxyapatite. In contrast, the successful application of the polymer coatings was confirmed by the emergence of supplementary sodium (Na) signals, associated with carboxymethyl cellulose, across all coated groups. Furthermore, the CMC-Ca-coated scaffolds exhibited an increased Ca signal relative to the CMC-only groups, a finding consistent with successful calcium-mediated crosslinking. The application of these polymer layers also produced a distinct surface-masking effect that significantly attenuated the detectable phosphorus (P) signal originating from the underlying 3DP-HA base. Consequently, the observed fluctuations in the Ca/P ratios do not indicate uniform bulk compositional changes, but rather reflect these specific surface-level modifications. The notably elevated Ca/P ratios seen in certain groups, such as 2CMC and 1:1 CMC-Ca, are primarily driven by the near-total masking of the underlying phosphorus, coupled with the localized surface deposition of the supplemental crosslinking calcium. Overall, these findings verify that the coating process effectively modified the surface elemental landscape of the scaffolds.

Scaffold porosity and pore architecture were characterized by micro-CT, and the resulting metrics are summarized in Table 3. Across all groups, total porosity remained in the range of approximately 54%–62%, with open porosity accounting for the majority of the total void volume, indicating that the coated scaffolds retained a predominantly interconnected pore structure. However, the slightly higher values observed in the 1% CMC-coated scaffolds may result from reduced coating continuity at this lower concentration, as well as from minor variations in coating thickness, both of which can influence porosity measurements (Sayed et al., 2018). Further optimization of the coating parameters could therefore improve uniformity and reduce measurement variability.

The mean pore diameters, as determined by micro-CT, ranged from approximately 23–28 µm. It should be noted that these pore diameters are smaller than the macroporosity range generally considered optimal for direct osteoblast infiltration and vascularized bone ingrowth (Cheng et al., 2014; Chen et al., 2020b; Jiao et al., 2023), which may be attributed to the resolution of the micro-CT scanning technique and the inherently small surface pores of the scaffolds. Consequently, the present porosity data are interpreted as evidence that the coating process preserved an interconnected porous architecture and did not occlude the existing pore network, rather than as direct confirmation of dimensions sufficient for cellular infiltration. The relationship between this pore architecture and osteoblast behavior was not directly assessed in the present study and would require complementary higher-resolution imaging and cell-infiltration analyses to establish.

All scaffold groups exhibited high blood absorption capacity, a property of clinical importance for the sequestration of platelets, fibrin, and endogenous growth factors (Kim et al., 2018). The absence of statistically significant differences among the groups indicates that the intrinsic macroporous architecture of the 3D-printed HA scaffolds predominantly governs blood absorption, with the surface coatings exerting little additional effect on overall fluid uptake. The hydrophilic nature of CMC, combined with calcium-induced ionic crosslinking, forms a stable three-dimensional hydrogel network with enhanced swelling and fluid-retention properties (Nan et al., 2019; Yao et al., 2024), which may contribute to the high absorption observed across the coated scaffolds. In addition, the incorporation of calcium could be advantageous for hemostasis, as calcium ions are essential cofactors in the coagulation cascade (Kim et al., 2018; Yao et al., 2024). Nevertheless, as the differences among groups were not statistically significant, no single formulation can be considered superior in terms of blood absorption, and the minor numerical variations most likely reflect experimental variability rather than a true coating effect. Overall, the comparable and high absorption capacity across all groups is a favorable outcome, indicating that the coatings are compatible with the functional demands of the scaffold while leaving the underlying blood-handling performance intact.

Both the qualitative and quantitative observations indicate that calcium-crosslinked CMC, particularly at the 2:1 ratio, is associated with a more favorable early clotting response under the conditions tested. The agreement between visible macroscopic clot formation and the lower BCI values is consistent with faster clot formation in these groups, although these two measures alone cannot characterize the individual steps of the coagulation cascade. It should also be noted that CaCl_2_ was supplied externally during the clotting assay. Consequently, the present data cannot fully separate the contribution of calcium derived from the CMC-Ca coating from that of the externally added calcium. The observed differences may therefore reflect surface chemistry, calcium made locally available by the coating, physical entrapment of blood components by the surface hydrogel, or a combination of these factors.

The improved clotting behavior may be related to the presence of calcium in the crosslinked coating, which could contribute to a localized calcium-enriched environment at the interface. Calcium ions are known cofactors in the coagulation cascade, including fibrin formation and platelet activation, and, consistent with previous reports (Zhao et al., 2024), available calcium may facilitate the formation of coagulation factor–calcium–phospholipid complexes and interactions between coagulation factors and platelet surfaces. In this respect, the proposed mechanism for the CMC-Ca coatings differs from that of several established hemostatic biomaterials. Kaolin, for example, promotes coagulation primarily by activating factor XII and the contact pathway through its negatively charged surface (Tamer et al., 2021), whereas chitosan-based dressings have been reported to enhance hemostasis through a largely clotting-factor–independent route, in which positively charged chitosan attracts and aggregates red blood cells to form a mucoadhesive barrier (Chen et al., 2020a). Beyond hemostasis, chitosan has also been used as a base for multifunctional scaffolds. For example, chitosan scaffolds incorporating Ti_2_NT_x_ MXene and carbon quantum dots have been reported to combine antibacterial and antioxidant activity with good hemocompatibility (Karatas et al., 2025). Collagen, by contrast, supports hemostasis by providing binding sites for platelet adhesion and activation and by promoting the aggregation of blood cells within its porous network (He et al., 2021). The calcium-associated mechanism proposed here is therefore distinct in emphasis, although the assays performed in the present study cannot establish which pathway predominates.

In addition, the hydrogel network formed on the scaffold surface may promote the physical entrapment of blood components, which could support cellular aggregation and clot retention (Zhao et al., 2024). This proposed contribution is broadly comparable to the porosity- and absorption-driven mechanisms described for collagen and other porous sponge dressings, in which rapid fluid uptake concentrates blood cells and coagulation factors at the material interface (He et al., 2021). These interpretations are proposed as possible contributing mechanisms. They are not directly demonstrated by the assays performed here, and confirmation would require additional analyses such as platelet adhesion/activation markers, higher-magnification imaging of the fibrin network, calcium-release measurements, and dynamic coagulation testing. The present coatings can also be compared with recent CMC- and calcium-based systems. Karatas et al. developed an injectable CaHA/CMC filler in which calcium hydroxyapatite microspheres were suspended in a CMC carrier, with no interaction between the mineral and CMC phases (Karatas et al., 2024). There, the calcium remained within a discrete mineral phase and the CMC acted mainly as a carrier, whereas in the present coatings calcium is ionically crosslinked to the CMC carboxylate groups at the surface. These represent contrasting strategies, namely, mineral encapsulation *versus* ionic complexation, which may place the calcium in different states of availability at the material–blood interface.

By comparison, the noncrosslinked CMC coatings, particularly at higher CMC content (2CMC), showed a transient delay in clotting relative to the CMC-Ca group. This behavior may be associated with the absence of added calcium in the coating and with poorer wettability, in which the outer layer rapidly forms a viscous hydrogel that can limit deeper fluid uptake and the contact of blood components with the surface, potentially slowing clot formation (Yao et al., 2024). A comparable concentration-dependent effect has been described for other hydrogel-forming polymers, where excessive surface viscosity can hinder the interaction between blood and the underlying material.

From an interfacial perspective, these blood–biomaterial interactions are fundamentally driven by surface free energy, wettability, and the work of adhesion governing blood constituents (Vijayanand et al., 2005). Upon blood contact, cell adhesion proceeds if it minimizes the net surface free energy of the system (Vijayanand et al., 2005). These physical parameters dictate a rapid redistribution of water, electrolytes, and plasma proteins via the Vroman effect, forming an initial nanometer-thick protein film that alters the 3D conformation of adsorbed clotting factors to guide platelet adhesion and cascade activation (De Mel et al., 2012). Consequently, the high polymer concentration in 2% CMC likely creates a highly viscous hydration layer that temporarily hinders this essential protein adsorption and subsequent cell adhesion. Conversely, the ionic complexation strategy of CMC-Ca coatings optimizes the surface energy profile and electrolyte availability, favorably altering the work of adhesion to accelerate macroscopic clot formation and cell retention.

Despite these differences in early clotting behavior, all scaffold groups ultimately supported clot formation, suggesting that the coatings did not adversely affect hemocompatibility under the conditions examined. The clotting response of the CMC-Ca coatings, combined with adequate blood uptake, supports clot formation on the scaffold surface (Yao et al., 2024). Because the clot provides an initial provisional matrix for subsequent cell recruitment, this may be relevant to the early stages of bone healing. However, the present study did not directly evaluate platelet behavior, fibrin architecture, calcium-release kinetics, or downstream osteogenic outcomes, and the mechanistic interpretation should therefore be regarded as preliminary.

From a biological perspective, cell attachment and early osteogenic activity are critical indicators of scaffold suitability (Thammarakcharoen et al., 2026). SEM observations at day 7 confirmed that surface modifications preserved a cell-adhesive environment. Cells exhibited a spread, flattened morphology with cytoplasmic extensions, indicating active interaction with the material surface. No qualitative differences in cell morphology or distribution were observed among coated and uncoated groups, suggesting that neither CMC nor CMC-Ca coating adversely affected early cell–material interactions. This aligns with the bio-adhesive nature of CMC (Mehrabi et al., 2024; Kargl et al., 2012), where the hydrated coating permits cell interaction with adsorbed proteins without limiting access to the underlying hydroxyapatite. In CMC–Ca-coated scaffolds, calcium incorporation did not induce cytotoxic effects or impaired adhesion, suggesting that the local calcium ions environment remained within a biologically tolerable range. These findings are consistent with previous studies demonstrating good cell adhesion and viability on CMC–HA hybrid systems (Teti et al., 2015).

ALP activity from this study is preliminary evidence of early osteogenic signaling and compatibility, showed no statistically significant differences among the CMC-coated, CMC-Ca-coated, and uncoated scaffolds after 7 days of incubation. Consistent with previous studies (Thammarakcharoen et al., 2026), a measurable, time-dependent increase in ALP expression on 3DP-HA surfaces was observed from day 7 onward. Although ALP levels in the scaffold groups were lower than those of the DMEM control, the CMC–Ca-coated groups showed no statistically significant difference from the control, suggesting preserved early osteoblastic function.

While this study demonstrates the potential of the proposed surface modification strategy, several limitations must be acknowledged. The material characterizations and biological assays reported here represent preliminary evidence of early osteogenic signaling and compatibility. The authors recognize that the sample size constitutes a limitation with respect to statistical power, particularly for high-variance biological and hematological metrics. Furthermore, this study addressed physical fluid interactions rather than providing a comprehensive confirmation of systemic hemocompatibility. Future work will incorporate quantitative coagulation assays, such as thromboelastography (Hartmann et al., 2023; Whitton and Healy, 2023) and markers of platelet activation (Yun et al., 2016), in order to comprehensively understand the role of these modifications in the hemostatic mechanism.

Moreover, the biological evaluation was limited to qualitative SEM observation of cell attachment and ALP activity at a single 7-day time point. Quantitative assays of cell viability, proliferation, and cytotoxicity were not performed, and therefore a comprehensive assessment of cytocompatibility could not be established. The present biological data should accordingly be regarded as preliminary, proof-of-concept evidence of early cell attachment and osteogenic signaling rather than a definitive confirmation of biological safety. Future studies will incorporate quantitative viability and proliferation assays, multiple culture time points, and long-term osteogenic gene expression analysis to fully establish the cytocompatibility and clinical potential of the modified scaffolds.

## 5 Conclusion

This preliminary study demonstrates the feasibility of using carboxymethyl cellulose (CMC) and calcium-crosslinked CMC (CMC–Ca) as tunable surface coatings on 3D-printed hydroxyapatite scaffolds to introduce early hemostatic functionality while preserving the interconnected porosity and early osteoblastic compatibility of the underlying framework. Under the conditions tested, all coated scaffolds retained total porosity between approximately 54% and 62% and comparable blood absorption capacity, while the 2:1 CMC–Ca formulation showed the most favorable early clotting tendency and did not impair cell attachment or day-7 ALP activity. These findings should be regarded as descriptive, proof-of-concept evidence rather than confirmation of hemostatic efficacy, as this study had several limitations, including the small sample size, single time point, and absence of quantitative viability and dynamic coagulation testing. Nonetheless, the results provide a coherent baseline supporting further development of CMC–Ca-modified scaffolds as multifunctional bone grafts, with future work incorporating platelet-activation and fibrin-network analyses, dynamic coagulation testing, calcium-release kinetics, quantitative cytocompatibility assays, and longer-term osteogenic markers.

## Data Availability

The original contributions presented in the study are included in the article/supplementary material, further inquiries can be directed to the corresponding author.
